# Bacterial Pneumonia and Acute Kidney Injury: Association and Impact on Outcomes of Patients Hospitalized for Acute Exacerbation of Chronic Obstructive Pulmonary Disease (COPD) in the United States

**DOI:** 10.2174/0118743064443250260210065237

**Published:** 2026-02-18

**Authors:** Thai Truong, Carlos Pichardo, Chanmi Park, Erick Phocco, Pedro Arias-Sanchez, Jeremy Kuszel, Lisa Glass

**Affiliations:** 1 Department of Medicine, St. Barnabas Hospital, 4422 Third Avenue, Bronx, NY 10457, USA; 2 Division of Pulmonary and Critical Care Medicine, St. Barnabas Hospital, 4422 Third Avenue, Bronx, NY 10457, USA

**Keywords:** Bacterial pneumonia, Acute kidney injury, Acute exacerbation of chronic obstructive pulmonary disease, Hospital mortality, COPD, Hospital length of stay, Endotracheal intubation

## Abstract

**Introduction:**

This study aims to investigate the association between bacterial pneumonia and acute kidney injury (AKI), which develops during hospitalizations for an acute exacerbation of chronic obstructive pulmonary disease (AECOPD), and their impact on patient outcomes.

**Methods:**

We performed a retrospective observational study on the United States National Inpatient Sample (USNIS) from 2016 to 2022, using ICD-10 codes to identify patients admitted to hospitals with an AECOPD who developed bacterial pneumonia and/or AKI during their hospital stay. We compared the clinical outcomes, including endotracheal intubation, in-hospital length of stay, and all-cause hospital mortality among four groups of patients: AECOPD, AECOPD with bacterial pneumonia and no AKI (PAECOPD), AECOPD with AKI and no bacterial pneumonia (KAECOPD), AECOPD with both bacterial pneumonia and AKI (PKAECOPD). We investigated the microorganism distribution of bacterial pneumonia and mortality by pathogen. We also used multivariate logistic and linear regression to investigate the correlation between outcomes and variables, including age, gender, race, hospital bed size, hospital location, bacterial pneumonia, AKI, and Charlson’s comorbidity index.

**Results:**

There were 2,548,188 weighted admissions, including 2,247,833 cases of AECOPD (88.21%); 34,930 cases of PAECOPD (1.37%); 258,360 cases of KAECOPD (10.14%); and 7,065 cases of PKAECOPD (0.28%). The average age of patients who died in hospitals was 6 years older than that of survivors (73.19 *vs* 67.70 years, *p*<0.01). Patients requiring endotracheal intubation were, on average, a year younger than those who did not (66.61 *vs* 67.77 years, *p*<0.01). White patients had poorer survival than Black, Hispanic, and other races. Females had lower hospital mortality than males by odds ratio (OR) 0.91 (*p*=0.004). AECOPD patients with bacterial pneumonia had a higher AKI rate than those without bacterial pneumonia (16.82% *vs* 10.31%, *p*<0.01). The PKAECOPD group had the poorest outcomes compared with the other groups, including higher endotracheal intubation incidence (27.18%), longer hospital stay (12.89 days), and higher all-cause hospital mortality (13.39%). Factors leading to increased all-cause hospital mortality included endotracheal intubation (OR 32.75, *p*<0.01), AKI (OR 2.33, *p*<0.01), bacterial pneumonia (OR 1.71, *p*<0.01), Charlson’s comorbidity index (OR 1.10, *p*<0.01), and older age (OR 1.05, *p*<0.01). Factors leading to increased hospital stay by at least a day included endotracheal intubation (6.13 days, *p*<0.01), bacterial pneumonia (3.03 days, *p*<0.01), and AKI (1.12 days, *p*<0.01). The most commonly identified pathogens causing bacterial pneumonia included other gram-negative bacilli (12.47%), *Pseudomonas aeruginosa* (11.54%), *Streptococcus pneumoniae* (7.22%), Methicillin-resistant *Staphylococcus aureus* (MRSA) (7.00%), *Mycoplasma pneumoniae* (4.70%), and *Hemophilus influenzae* (4.63%). In approximately forty percent of cases, no specific pathogen was identified. Mortality was highest for patients with “other bacteria” (23.43%), MRSA (22.86%), and *Pseudomonas aeruginosa* (20%).

**Discussion:**

Patients admitted with an AECOPD had a high incidence of AKI during hospital admission, approximately 10%. Patients with an AECOPD who developed bacterial pneumonia represented a small proportion of admissions (1.65%) but had a higher risk of AKI (16.82%). These patients were likely to be infected with pathogens including *Pseudomonas aeruginosa*, other gram-negative bacteria, and MRSA. Patients with both AKI and bacterial pneumonia had the highest all-cause hospital mortality.

**Conclusion:**

Our study found that hospitalized AECOPD patients with bacterial pneumonia had a higher rate of AKI than those without bacterial pneumonia, and the population of AECOPD patients with both bacterial pneumonia and AKI had markedly higher all-cause hospital mortality, longer hospital stays, and greater need for endotracheal intubation. Therefore, minimizing the association between bacterial pneumonia and AKI may help improve the prognosis of patients admitted with an AECOPD.

## 1 INTRODUCTION

COPD is the sixth leading cause of death in the United States in 2020 [1]. It is characterized by abnormalities of the lower airways (bronchitis, bronchiolitis) and/or alveoli (emphysema), which cause persistent and often progressive airflow obstruction [2]. Patients with COPD present with chronic respiratory symptoms, including dyspnea and productive cough with sputum, which limits their activity [2]. Acute exacerbation of COPD is an event characterized by an acute increase in dyspnea, cough, and/or sputum production over 14 days or fewer. It may be accompanied by tachypnea and/or tachycardia and is often associated with increased local and systemic inflammation caused by infection, pollution, or other insults to the airways [3].

Bacterial pneumonia and acute kidney injury (AKI) are common in AECOPD patients [4-8]. They are associated with worse clinical outcomes including mortality, hospital length of stay (LOS), mechanical ventilation, ICU admission, and hospital readmission [4-20]. However, to our knowledge, the association of these two clinical conditions and the impact of this association on the outcomes of the AECOPD population are not well studied. In addition, most prior studies did not specify whether they included bacterial or viral pneumonia, and even fewer investigated the bacterial distribution of pneumonia in AECOPD patients. Therefore, we performed this study on the United States National Inpatient Sample to investigate the association of bacterial pneumonia and AKI that developed during hospitalization for an AECOPD. We also studied the outcomes of AECOPD patients who developed bacterial pneumonia and/or AKI, including endotracheal intubation requirements, hospital LOS, and all-cause hospital mortality. Furthermore, we aimed to provide the distribution of pathogens in bacterial pneumonia and the mortality rate by pathogen in patients with an AECOPD.

## 2 MATERIALS AND METHODS

### 2.1 Materials

The USNIS is the largest publicly available inpatient database covering more than 97% of the United States population. It is developed for the Healthcare Cost and Utilization Project under the sponsorship of the Agency for Healthcare Research and Quality. It contains a stratified sample, which is equivalent to 7 million (unweighted) or 35 million (weighted) hospital admissions per year from community hospitals in 47 states and the District of Columbia. The database excludes rehabilitation and long-term care facilities. The USNIS protects patient confidentiality because it does not provide personal, state, or hospital identification. Since 2016, USNIS has coded diagnoses and procedures by the International Classification of Diseases, Tenth Revision, Clinical Modification/Procedure Coding System (ICD-10-CM/PCS) [21].

### 2.2 Methods

**Fig. (1):**
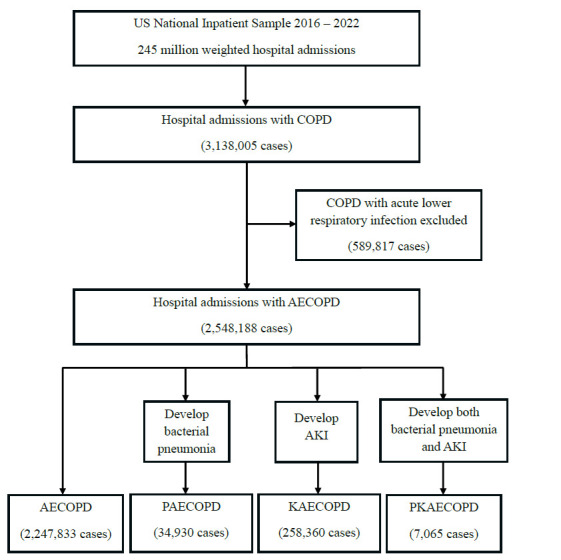
Flow diagram.

We performed a retrospective cohort study on the USNIS for 7 years from January 01, 2016, to December 31, 2022 (Fig. **1**). The ICD-10 code was used to identify patients (Table **1**). Participants were at least 18 years old at the time of admission with a principal diagnosis of AECOPD. We excluded patients with a principal diagnosis of COPD with acute lower respiratory infection, as this group of patients might have pneumonia, acute bronchitis, acute bronchiolitis, or influenza at the time of hospital admission. We then identified AECOPD patients with a secondary diagnosis of bacterial pneumonia and/or AKI. Patients were categorized into four groups: AECOPD with no bacterial pneumonia and no AKI (AECOPD), AECOPD with bacterial pneumonia and no AKI (PAECOPD), AECOPD with AKI and no bacterial pneumonia (KAECOPD), AECOPD with both bacterial pneumonia and AKI (PKAECOPD). We compared primary outcomes, including need for endotracheal intubation, hospital length of stay, and all-cause hospital mortality between the four groups. Bacterial pathogens and mortality rates by each pathogen were identified. Other identified diagnoses included lung cancer, cardiovascular diseases, pulmonary embolism, and liver cirrhosis. Cardiovascular diseases are a combination of essential hypertension, congestive heart failure, pulmonary edema, myocardial infarction, acute coronary thrombosis, atrial fibrillation, and ventricular fibrillation.

Stata 18.5 (StataCorp, College Station, Texas) was used for statistical analysis. Categorical variables are presented as weighted numbers with percentages, and qualitative variables are presented as mean values with 95% confidence intervals (CI). An independent samples t-test was used to compare mean values, and Pearson’s chi-square test was used to compare categorical variables. We investigated the correlation of all-cause hospital mortality with bacterial pneumonia, AKI, Charlson’s comorbidity index, gender, age, race, hospital bed size, and hospital location by multivariate logistic regression. We also investigated the correlation of hospital LOS with the above factors by multivariate linear regression. This study did not require informed consent or institutional review board approval because it is an observational study based on a codified confidential sample of patients with no hospital or patient identifiers provided.

## 3 RESULTS

### 3.1 Outcomes by Groups of Patients

Among 3,138,005 weighted hospital admissions with a principal diagnosis of COPD, we excluded 589,817 cases of COPD with acute lower respiratory infection and included 2,548,188 cases of AECOPD. Of these cases, there were 2,247,833 cases of AECOPD with no bacterial pneumonia and no AKI (AECOPD, 88.21%); 34,930 cases of AECOPD with bacterial pneumonia and no AKI (PAECOPD, 1.37%); 258,360 cases of AECOPD with AKI and no bacterial pneumonia (KAECOPD, 10.14%); and 7,065 cases of AECOPD with both bacterial pneumonia and AKI (PKAECOPD, 0.28%) (Table **2**). Patients with bacterial pneumonia had a higher rate of AKI than those without bacterial pneumonia (16.82% *vs* 10.31%, *p*<0.01) (Table **3**). Patients with KAECOPD had the highest mean age (71.15 years old, 95% CI 71.04-71.25), followed by patients with PKAECOPD (70.39 years old, 95% CI 69.82-70.96), PAECOPD (68.29 years old, 95%CI 68.03-68.56), and AECOPD (67.35 years old, 95%CI 67.29-67.40). In all four groups, more females were present. By race, in descending order, were White, Black, Hispanic, Asian/Pacific Islander, and Native American patients.

Patients who died in hospital from all causes were about 6 years older than survivors (73.19 *vs* 67.70 years old, *p*<0.01). Patients who were intubated were younger than those who were not intubated (66.61 *vs* 67.77 years old, *p*<0.01). Patients from PKAECOPD group had the highest rate of endotracheal intubation and all-cause hospital mortality rate (27.18% for intubation, 13.39% for mortality) compared with KAECOPD (5.03% for intubation, 3.09% for mortality), PAECOPD (7.59% for intubation, 2.30% for mortality), and AECOPD (1.33% for intubation, 0.70% for mortality), *p*<0.01. The mean hospital LOS was also longest in the PKAECOPD group (12.89 days, 95%CI 12.04-13.74); followed by PAECOPD (6.71 days, 95%CI 6.54-6.88); KAECOPD (5.37 days, 95%CI 5.33-5.42); and AECOPD (3.85 days, 95%CI 3.83-3.86); *p*<0.01.

### 3.2 Outcomes by Bacterial Pathogen in Patients who Developed Bacterial Pneumonia

Among the specified pathogens, the six most common microorganisms were other gram-negative bacilli (12.47%), *Pseudomonas aeruginosa* (11.54%), *Streptococcus pneumoniae* (7.22%), MRSA (7.00%), *Mycoplasma pneumoniae* (4.70%), and *Hemophilus influenzae* (4.63%). Among deaths in the bacterial pneumonia group, the highest mortality rates were from other bacteria (23.43%), MRSA (22.86%), and *Pseudomonas aeruginosa* (20%). Regarding death per pathogens, MRSA and MSSA had the highest rate of mortality (13.61% and 12.96%, respectively). Other bacteria include those species that do not have a specific ICD-10 code (Table **4**).

### 3.3 Factors Affecting Outcomes

Patients who were intubated had a significantly higher hospital mortality rate than those who were not intubated: 14.5% *vs* 0.51% in AECOPD, 13.77% *vs* 1.36% in PAECOPD, 28.51% *vs* 1.75% in KAECOPD, 28.65% *vs* 7.68% in PKAECOPD; *p*<0.01. Factors significantly increased the mortality of hospitalization for an AECOPD, including endotracheal intubation (OR 32.75, *p*<0.01), AKI (OR 2.33, *p*<0.01), bacterial pneumonia (OR 1.71, *p*<0.01), Charlson’s comorbidity index (OR 1.10, *p*<0.01), and older age (OR 1.05, *p*<0.01). Females had lower mortality than males, OR 0.91 (*p*=0.004). Black, Hispanic, Asian/Pacific Islander, and other races had lower mortality compared to White patients by OR 0.59 (*p*<0.01), 068 (*p*<0.01), 0.73 (*p*=0.030), and 0.75 (*p*=0.019), respectively. Compared with patients admitted to rural hospitals, those admitted to urban non-teaching and urban teaching hospitals had lower mortality by OR 0.87 (*p*=0.007) and 0.91 (*p*=0.048) (Tables **5** and **6**).

Factors significantly increased hospital LOS by at least one more day, including endotracheal intubation (6.13 days, *p*<0.01), bacterial pneumonia (3.03 days, *p*<0.01), and AKI (1.12 days, *p*<0.01). Black, Hispanic, Asian/Pacific Islander, and Native American patients had shorter hospital LOS than White patients by 0.14 (*p*<0.01), 0.06 (*p*=0.010), 0.18 (*p*=0.001), and 0.17 (*p*=0.028) days, respectively. Patients in medium and large bed-size hospitals stayed in hospitals shorter than those in small bed-size hospitals by 0.19 and 0.36 days, respectively (*p*<0.01). Patients in urban non-teaching and urban teaching hospitals had shorter hospital LOS than those in rural hospitals by 0.34 and 0.50 days, respectively (*p*<0.01).

## 4 DISCUSSION

The incidence of pneumonia in patients hospitalized for an AECOPD varies from 16-36% in prior studies [8, 9, 19]. Pneumonia is commonly diagnosed by chest imaging and clinical findings without waiting for respiratory culture results. In our study, we only included bacterial pneumonia with identified pathogens developed during hospital stay. Therefore, the incidence of bacterial pneumonia in our study is 1.65% and lower than in previous studies. The most common bacterial pathogens found in an AECOPD are *Hemophilus influenzae*, *Moraxella catarrhalis, Streptococcus pneumoniae*, and *Pseudomonas aeruginosa* [22]. Two studies showed that in the COPD population with community-acquired pneumonia, *Streptococcus pneumoniae* was the most common (6.5%-26%), followed by *Pseudomonas aeruginosa* (5.6%), *Hemophilus influenzae* (3.7%), *Staphylococcus aureus* (3.2%), and *Legionella pneumophila* (2%) [23, 24]. Another study by Shin *et al* also found *Streptococcus pneumoniae* remained the most common pathogen (40.3%), followed by *Staphylococcus aureus* (14.2%), *Pseudomonas aeruginosa* (12.7%), and *Klebsiella pneumoniae* (5.2%) in patients who developed community-acquired pneumonia in the setting of AECOPD [15]. However, our study found *Pseudomonas aeruginosa* is the most commonly identified pathogen (11.54%), which outnumbers *Streptococcus pneumoniae* (7.22%), MRSA (7.00%), and *Hemophilus influenzae* (4.63%). MRSA and *Pseudomonas aeruginosa* infections had the highest mortality rate (22.86% and 20%, respectively). The presence of these organisms may suggest recurrent hospital admissions or healthcare exposure in the COPD population in the United States. The higher incidence of *P*se*udomonas aeruginosa* infection may reflect the incidence of structural lung diseases, including emphysematous changes. There were 41,995 cases of bacterial pneumonia, and 43,650 bacterial pathogens were isolated from respiratory cultures (Tables **3** and **4**). This indicates that many cases of bacterial pneumonia involved more than one isolated bacterial pathogen. However, we were not able to identify these multi-bacterial pneumonia cases due to NIS limitations. In addition, as many uncommon bacteria were isolated from respiratory cultures, several lacked unique ICD-10 codes. Instead, they are classified by ICD-10 as “other specified bacteria” or “unspecified bacteria.” For this reason, we could not precisely identify bacteria in these two groups; therefore, we combined them into a common group labeled as “other” bacteria (Table **4**). These other bacteria accounted for 45.76% of the total pathogens and 23.43% of hospital deaths.

Inhaled corticosteroid (ICS) use may increase the risk for pneumonia in patients with COPD [16, 25]. Alternatively, prior ICS use was found to decrease endotracheal intubation, mechanical ventilation, and short-term mortality in the COPD population hospitalized for pneumonia [26]. According to a review by Yu *et al*, patients with COPD and pneumonia are less likely to have prior ICS use than those without pneumonia [20]. The mixed results from previous studies allow for various interpretations of the effect of ICS. This is further compounded by guidelines for COPD management, which changed the indication for ICS in COPD to patients with serum eosinophilia during the study period [27]. In a meta-analysis, pneumonia was associated with higher in-hospital mortality (relative risk [RR] 2.29), longer hospital LOS (weighted mean difference 3.31), increased mechanical ventilation (RR 2.02), and more ICU admissions (RR 2.79) in patients with AECOPD [16]. The requirement for invasive mechanical ventilation and hospital mortality rate of AECOPD patients with pneumonia varied 1.2%-17.4% and 3.8%-42% respectively in multiple studies [8, 9, 13-15, 17-19, 28-32]. This large variation is attributed to the varying severity of baseline COPD, co-morbid conditions, and healthcare system quality. In our study, the group of patients with an AECOPD and pneumonia had similar outcomes to the above studies: 4.17% died in hospital and 10.88% required intubation. In addition, mortality rates vary among different racial groups. Our study found that White patients were the largest population and had worse outcomes than patients of other races.

AKI occurs in 1.90–27.50% of patients with AECOPD [4-6, 10-12, 33]. According to Wan *et al*., the group of AECOPD patients with AKI has a higher endotracheal intubation rate (18.3% *vs*. 3.1%), a higher in-hospital mortality rate (18.0% *vs*. 2.7%), and longer in-hospital LOS (13 days *vs*. 10 days) than the group without AKI [5]. Stage 3 AKI increases the risk of in-hospital mortality up to 6.0-fold [5]. In our study, the AKI rate in patients with an AECOPD was 10.31%, and the intubation rate in this group was 5.03%. However, if they developed bacterial pneumonia, the AKI and intubation rates were significantly higher at 16.82% and 10.88%, respectively. Patients with bacterial pneumonia are more likely to develop complications requiring medications with potential nephrotoxic effects (*e.g*., antibiotics, vasopressors), which increase the risk of AKI. In addition, the correlation between respiratory and kidney failure was further explained by several pathophysiological mechanisms: (a) hypoxic respiratory failure directly diminishes renal blood flow, while hypercapnic respiratory failure activates renal vasoconstriction and systemic vasodilation, resulting in lower renal blood flow [12, 34-36]; (b) systemic proinflammatory mediators from acute respiratory failure are associated with AKI development and vice versa [12, 37-39]; (c) hemodynamic disturbances, blood gas impairment, neurohumoral alterations, and bio-trauma induced by mechanical ventilation reduce renal function [12, 34, 36, 40]; (d) high intra-abdominal pressure induced by COPD and mechanical ventilation decreases venous return, resulting in renal edema and failure [12, 36, 37]; (e) lower expression of pulmonary-predominant water channels and aquaporin 5 due to AKI may damage lung tissues [12, 41].

Although bacterial pneumonia is associated with a higher AKI rate, only 0.28% of patients with an AECOPD developed both bacterial pneumonia and AKI in our study. This group of patients, however, had markedly worse outcomes. The mortality rates for AECOPD, PAECOPD, and KAECOPD were 0.70%, 2.30%, and 3.09%, respectively. When both bacterial pneumonia and AKI were present, the mortality rate increased to 13.39%. Patients in this group had an average hospital LOS of 12.89 days, which is significantly longer than in the other groups. Endotracheal intubation, an indicator of critical illness, was performed in 27.18% of patients in this group, significantly prolonging hospital LOS by 6.13 days and increasing hospital mortality (OR 32.75).

## 5 LIMITATIONS

Our study did not identify the timing of pneumonia onset because we included pneumonia cases based on pathogens defined by ICD-10 codes. Therefore, we could not distinguish hospital-acquired pneumonia from community-acquired pneumonia. We also could not identify uncommon bacterial pathogens or multi-bacterial pneumonia cases. In addition, we did not investigate the baseline severity of COPD before admission. Patients with a history of COPD exacerbation have a higher risk of future exacerbations; however, the USNIS dataset is unable to identify patients with recurrent admissions. As our study is retrospective and observational, there are residual confounders that may impact the outcomes, *e.g*., non-coded diagnoses, time of year, comorbid viral illnesses, repeat admissions of the same patient, and so on.

Body fluid status is an important determinant of outcomes in patients with acute kidney injury. However, since NIS does not provide data on fluid intake and output, intravenous fluid administration, or daily diuretic therapy, we could not evaluate the body fluid status of patients with acute kidney injury in our study. Also unknown is the severity of AKI and the presence of underlying chronic kidney disease due to USNIS limitations.

## CONCLUSION

Hospitalized patients with an AECOPD who developed bacterial pneumonia had a higher rate of AKI. Although patients with both conditions accounted for only 0.28% of cases, they had significantly worse outcomes, including longer hospital stays, higher rates of endotracheal intubation, and increased all-cause hospital mortality. Therefore, minimizing the association between bacterial pneumonia and AKI may help improve the prognosis of patients admitted with an AECOPD. Focusing on all-cause mortality, a clinically and epidemiologically important endpoint, may explain the differences from prior studies.

## Figures and Tables

**Table 1:** ICD-10 clinical modification/procedure coding system for variables.

**ICD-10 CM/PCS Code**	**Clinical Condition or Procedure**
J 44.1	Acute exacerbation of COPD
J13	Pneumonia due to *Streptococcus pneumoniae*
J14	Pneumonia due to *Hemophilus influenzae*
J15.0	Pneumonia due to *Klebsiella pneumoniae*
J15.1	Pneumonia due to *Pseudomonas aeruginosa*
J15.211	Pneumonia due to MSSA
J15.212	Pneumonia due to MRSA
J15.3	Pneumonia due to *Streptococcus group B*
J15.4	Pneumonia due to other Streptococci
J15.5	Pneumonia due to *Escherichia coli*
J15.6	Pneumonia due to other gram-negative bacteria
J15.7	Pneumonia due to *Mycoplasma pneumoniae*
J15.8	Pneumonia due to other specified bacteria
J15.9	Pneumonia due to unspecified bacteria
J16.0	Pneumonia due to *Chlamydia pneumoniae*
C34	Lung cancer
I26, I 26.0, I26.9, I27.82	Pulmonary embolism
N17	Acute kidney injury
K70, K74, K71.7	Liver cirrhosis
(*)	Cardiovascular diseases
0BH17EZ	Endotracheal intubation

**Note: **(*): Cardiovascular diseases include essential hypertension (I10), congestive heart failure (I50, I110), pulmonary edema (J81, J81.0, J81.1), myocardial infarction (I21, I22), acute coronary thrombosis (I24.0), atrial fibrillation (I48), and ventricular fibrillation (I49.0, I49.01, I49.02).

**Table 2:** Outcomes, comorbidities, and demographics of four groups of patients.

**Variables**	**AECOPD**	**PAECOPD**	**KAECOPD**	**PKAECOPD**	***P*-value**
Admission, N (%)	2,247,833 (88.21)	34,930 (1.37)	258,360 (10.14)	7,065 (0.28)	
Hospital mortality, N (%)	15,695 (0.70)	805 (2.30)	7,995 (3.09)	945 (13.39)	< 0.01
Endotracheal intubation, N (%)	29,830 (1.33)	2,650 (7.59)	13,005 (5.03)	1,920 (27.18)	< 0.01
Hospital LOS, days (95%CI)	3.85 (3.83-3.86)	6.71 (6.54-6.88)	5.37 (5.33-5.42)	12.89 (12.04-13.74)	< 0.01
Lung cancer, N (%)	11,062 (2.46)	242 (3.46)	1,182 (2.29)	45 (3.18)	< 0.01
Cardiovascular diseases, N (%)	333,655 (74.22)	5,144 (73.63)	40,324 (78.04)	1,153 (81.60)	< 0.01
Pulmonary embolism, N (%)	854 (0.19)	12 (0.17)	101 (0.20)	4 (0.28)	0.753
Liver cirrhosis, N (%)	7,073 (1.57)	112 (1.60)	1,056 (2.04)	24 (1.70)	0.034
Females, N (%)	1,317,609 (58.62)	19,345 (55.38)	132,750 (51.38)	3,570 (50.53)	< 0.01
Mean age, year (95% CI)	67.35 (67.29-67.40)	68.29 (68.03-68.56)	71.15 (71.04-71.25)	70.39 (69.82-70.96)	0.005
Race, N (%)					
-White	1,712,849 (76.20)	28,730 (82.25)	180,852 (70.00)	5,235 (74.10)	< 0.01
-Black	340,996 (15.17)	3,269 (9.36)	51,853 (20.07)	1,166 (16.50)	
-Hispanic	119,135 (5.30)	1,666 (4.78)	15,734 (6.10)	430 (6.08)	
-Asian/Pacific Islander	23,602 (1.05)	445 (1.27)	3,695 (1.43)	92 (1.30)	
-Native American	12,611 (0.56)	221 (0.63)	1,240 (0.47)	9 (0.13)	
-Other	38,640 (1.72)	599 (1.71)	4,986 (1.93)	133 (1.88)	

**Table 3:** AKI rate and outcome difference between AECOPD without and with bacterial pneumonia.

-	**AECOPD without Bacterial Pneumonia**	**AECOPD with Bacterial Pneumonia**	***P*-value**
N (%)	2,506,193 (98.35)	41,995 (1.65)	-
AKI, N (%)	258,388 (10.31)	7,064 (16.82)	<0.01
Endotracheal intubation, N (%)	42,856 (1.71)	4,569 (10.88)	<0.01
Hospital LOS, days (95%CI)	4.00 (3.99-4.01)	7.75 (7.54-7.95)	<0.01
Hospital mortality, N (%)	23,690 (0.95)	1,750 (4.17)	<0.01

**Table 4:** Pathogen distribution and mortality rate in the bacterial pneumonia group.

**Bacterial Pneumonia Pathogens**	**N (%)**	**Mortality per Pathogens N (%)**	**Mortality per Total Death Toll, %**
*Streptococcus pneumoniae*	3,030 (7.22)	100 (3.30)	5.71
*Hemophilus influenzae*	1,945 (4.63)	35 (1.80)	2.00
*Klebsiella pneumoniae*	1,110 (2.64)	90 (8.11)	5.14
*Pseudomonas aeruginosa*	4,845 (11.54)	350 (7.22)	20.00
MSSA	1,080 (2.57)	140 (12.96)	8.00
MRSA	2,940 (7.00)	400 (13.61)	22.86
*Streptococcus group B*	80 (0.19)	0	0
Other Streptococci	1,495 (3.56)	20 (1.34)	1.14
*Escherichia coli*	650 (1.55)	40 (6.15)	2.28
Other gram-negative bacteria	5,235 (12.47)	290 (5.54)	16.57
*Mycoplasma pneumoniae*	1,975 (4.70)	25 (1.27)	1.43
*Chlamydia pneumoniae*	50 (0.12)	0	0
Other (*)	19,215 (45.76)	410 (2.13)	23.43

**Note:** (*): the total of “other specified bacteria” and “unspecified bacteria”.

**Table 5:** Impact of factors on overall hospital mortality and length of stay.

**Variables**	**Hospital Mortality**		**Hospital Length of Stay**	
	**Odds Ratio**	***P*-value**	**Coefficient**	***P*-value**
Endotracheal intubation	-	-	-	-
-No	Reference	-	Reference	-
-Yes	32.75	<0.01	6.13	<0.01
Gender	-	-	-	-
-Male	Reference	-	Reference	-
-Female	0.91	0.004	0.29	<0.01
Age	1.05	<0.01	0.01	<0.01
Race	-	-	-	-
-White	Reference	-	Reference	-
-Black	0.59	<0.01	-0.14	<0.01
-Hispanic	0.68	<0.01	-0.06	0.01
-Asian/Pacific Islander	0.73	0.03	-0.18	0.001
-Native Americans	1.22	0.359	-0.17	0.028
-Other	0.75	0.019	0.02	0.594
Hospital bed size	-	-	-	-
-Small	Reference	-	Reference	-
-Medium	1.02	0.702	0.19	<0.01
-Large	1.07	0.087	0.36	<0.01
Hospital location	-	-	-	-
-Rural	Reference	-	Reference	-
-Urban non-teaching	0.87	0.007	0.34	<0.01
-Urban teaching	0.91	0.048	0.5	<0.01
Bacterial pneumonia	-	-	-	-
-No	Reference	-	Reference	-
-Yes	1.71	<0.01	3.03	<0.01
AKI	-	-	-	-
-No	Reference	-	Reference	-
-Yes	2.33	<0.01	1.12	<0.01
Charlson’s comorbidity index	1.1	<0.01	0.13	<0.01

**Table 6:** Hospital mortality between groups with and without endotracheal intubation.

-	**Endotracheal Intubation**	**No Endotracheal Intubation**	***P*-value**
AECOPD, N (%)	4,325 (14.50)	11,312 (0.51)	<0.01
PAECOPD, N (%)	365 (13.77)	439 (1.36)	<0.01
KAECOPD, N (%)	3,708 (28.51)	4,294 (1.75)	<0.01
PKAECOPD, N (%)	550 (28.65)	395 (7.68)	<0.01

## Data Availability

The data supporting the findings of this article are available in the Agency for Healthcare Research and Quality’s (AHRQ) Healthcare Cost and Utilization Project (HCUP) National Inpatient Sample (NIS) repository https://hcup-us.ahrq.gov/nisoverview.jsp. Access requires completion of the HCUP Data Use Agreement and purchase of the dataset.

## LIST OF ABBREVIATIONS

AECOPD: Acute exacerbation of chronic obstructive pulmonary disease
AKI: Acute kidney injury
CI: Confidence Interval
CM/PCS: Clinical Modification/Procedure Coding System
COPD: Chronic obstructive pulmonary disease
ICD-10: International Classification of Diseases-10
ICS: Inhaled corticosteroid
KAECOPD: Acute exacerbation of chronic obstructive pulmonary disease with acute kidney injury and no bacterial pneumonia
LOS: length of stay
MRSA: Methicillin-resistant *Staphylococcus aureus*
MSSA: Methicillin-sensitive *Staphylococcus aureus*
NIS: National Inpatient Sample
OR: Odds ratio
PAECOPD: Acute exacerbation of chronic obstructive pulmonary disease with bacterial pneumonia and no acute kidney injury
PKAECOPD: Acute exacerbation of chronic obstructive pulmonary disease with both bacterial pneumonia and acute kidney injury
RR: Relative risk
USNIS: United States National Inpatient Sample

## References

[1] Syamlal G., Kurth L.M., Dodd K.E., Blackley D.J., Hall N.B., Mazurek J.M. (2022). Chronic obstructive pulmonary disease mortality by industry and occupation — United States, 2020.. MMWR Morb. Mortal. Wkly. Rep..

[2] Celli B., Fabbri L., Criner G., Martinez F.J., Mannino D., Vogelmeier C., Montes de Oca M., Papi A., Sin D.D., Han M.K., Agusti A. (2022). Definition and nomenclature of chronic obstructive pulmonary disease: Time for its revision.. Am. J. Respir. Crit. Care Med..

[3] Celli B.R., Fabbri L.M., Aaron S.D., Agusti A., Brook R., Criner G.J., Franssen F.M.E., Humbert M., Hurst J.R., O’Donnell D., Pantoni L., Papi A., Rodriguez-Roisin R., Sethi S., Torres A., Vogelmeier C.F., Wedzicha J.A. (2021). An updated definition and severity classification of chronic obstructive pulmonary disease exacerbations: The Rome Proposal.. Am. J. Respir. Crit. Care Med..

[4] Barakat M., McDonald H., Collier T., Smeeth L., Nitsch D., Quint J. (2015). Acute kidney injury in stable COPD and at exacerbation.. Int. J. Chron. Obstruct. Pulmon. Dis..

[5] Wan X., Chen D., Tan Y., Ma M., Zhang F., Liu Z., Chen Y., Shao W., Cao C. (2020). incidence, risk factors, and prognostic implications of acute kidney injury in patients with acute exacerbation of COPD.. Int. J. Chron. Obstruct. Pulmon. Dis..

[6] Cao C. C., Chen D. W., Li J., Ma M. Q., Chen Y. B., Cao Y. Z., Hua X., Shao W., Wan X. (2018). Community-acquired *versus* hospital-acquired acute kidney injury in patients with acute exacerbation of COPD requiring hospitalization in China.. Int. J. Chron. Obstruct. Pulmon. Dis..

[7] Niu Y., Xing Y., Li J., Shui W., Gu Y., Zhang C., Du H. (2021). Effect of community-acquired pneumonia on acute exacerbation of chronic obstructive pulmonary disease.. COPD.

[8] Søgaard M., Madsen M., Løkke A., Hilberg O., Sørensen H. T., Thomsen R. W. (2016). Incidence and outcomes of patients hospitalized with COPD exacerbation with and without pneumonia.. Int. J. Chron. Obstruct. Pulmon. Dis..

[9] Myint P.K., Lowe D., Stone R.A., Buckingham R.J., Roberts C.M. (2011). U.K. National COPD Resources and Outcomes Project 2008: patients with chronic obstructive pulmonary disease exacerbations who present with radiological pneumonia have worse outcome compared to those with non-pneumonic chronic obstructive pulmonary disease exacerbations.. Respiration.

[10] Hirayama A., Goto T., Hasegawa K. (2020). Association of acute kidney injury with readmissions after hospitalization for acute exacerbation of chronic obstructive pulmonary disease: A population-based study.. BMC Nephrol..

[11] Fabbian F., De Giorgi A., Manfredini F., Lamberti N., Forcellini S., Storari A., Gallerani M., Caramori G., Manfredini R. (2016). Impact of renal dysfunction on in-hospital mortality of patients with severe chronic obstructive pulmonary disease: a single-center Italian study.. Int. Urol. Nephrol..

[12] Chen D., Jiang L., Li J., Tan Y., Ma M., Cao C., Zhao J., Wan X. (2021). Interaction of acute respiratory failure and acute kidney injury on in-hospital mortality of patients with acute exacerbation COPD.. Int. J. Chron. Obstruct. Pulmon. Dis..

[13] Lu Z., Cheng Y., Tu X., Chen L., Chen H., Yang J., Wang J., Zhang L. (2016). Community-acquired pneumonia and survival of critically ill acute exacerbation of COPD patients in respiratory intensive care units.. Int. J. Chron. Obstruct. Pulmon. Dis..

[14] Steer J., Norman E.M., Afolabi O.A., Gibson G.J., Bourke S.C. (2012). Dyspnoea severity and pneumonia as predictors of in-hospital mortality and early readmission in acute exacerbations of COPD.. Thorax.

[15] Shin B., Kim S.H., Yong S.J., Lee W.Y., Park S., Lee S.J., Lee S.J., Lee M.K. (2019). Early readmission and mortality in acute exacerbation of chronic obstructive pulmonary disease with community-acquired pneumonia.. Chron. Respir. Dis..

[16] Zheng F., Wang X. (2024). Effect of pneumonia on the outcomes of acute exacerbation of chronic obstructive pulmonary disease: a systematic review and meta-analysis.. BMC Pulm. Med..

[17] Andreassen S.L., Liaaen E.D., Stenfors N., Henriksen A.H. (2014). Impact of pneumonia on hospitalizations due to acute exacerbations of COPD.. Clin. Respir. J..

[18] Sharafkhaneh A., Spiegelman A.M., Main K., Tavakoli-Tabasi S., Lan C., Musher D. (2017). Mortality in patients admitted for concurrent COPD exacerbation and pneumonia.. COPD.

[19] Saleh A., López-Campos J.L., Hartl S., Pozo-Rodríguez F., Roberts C.M., European COPD Audit team (2015). The effect of incidental consolidation on management and outcomes in COPD Exacerbations: Data from the European COPD Audit.. PLoS One.

[20] Yu Y., Liu W., Jiang H.L., Mao B. (2021). Pneumonia is associated with increased mortality in hospitalized COPD patients: A systematic review and meta-analysis.. Respiration.

[21] Healthcare Cost and Utilization Project (2025). Healthcare cost and utilization project, overview of the National (Nationwide) Inpatient Sample.. https://hcup-us.ahrq.gov/nisoverview.jsp.

[22] Sethi S. (2004). Bacteria in exacerbations of chronic obstructive pulmonary disease: phenomenon or epiphenomenon?. Proc. Am. Thorac. Soc..

[23] Restrepo M.I., Mortensen E.M., Pugh J.A., Anzueto A. (2006). COPD is associated with increased mortality in patients with community-acquired pneumonia.. Eur. Respir. J..

[24] Molinos L., Clemente M.G., Miranda B., Alvarez C., del Busto B., Cocina B.R., Alvarez F., Gorostidi J., Orejas C., ASTURPAR Group (2009). Community-acquired pneumonia in patients with and without chronic obstructive pulmonary disease.. J. Infect..

[25] Ernst P., Gonzalez A.V., Brassard P., Suissa S. (2007). Inhaled corticosteroid use in chronic obstructive pulmonary disease and the risk of hospitalization for pneumonia.. Am. J. Respir. Crit. Care Med..

[26] Chen D., Restrepo M.I., Fine M.J., Pugh M.J., Anzueto A., Metersky M.L., Nakashima B., Good C., Mortensen E.M. (2011). Observational study of inhaled corticosteroids on outcomes for COPD patients with pneumonia.. Am. J. Respir. Crit. Care Med..

[27] Global Initiative for Chronic Obstructive Lung Disease (2025). Global strategy for the diagnosis, management, and prevention of chronic obstructive pulmonary disease (2025 Report).. https://goldcopd.org/2025-gold-report/.

[28] Boixeda R., Bacca S., Elias L., Capdevila J.A., Vilà X., Mauri M., Almirall J. (2014). La neumonía como comorbilidad en la enfermedad pulmonar obstructiva crónica (EPOC). Diferencias entre la exacerbación aguda de la EPOC y la neumonía en los pacientes con EPOC.. Arch. Bronconeumol..

[29] Huerta A., Crisafulli E., Menéndez R., Martínez R., Soler N., Guerrero M., Montull B., Torres A. (2013). Pneumonic and nonpneumonic exacerbations of COPD: inflammatory response and clinical characteristics.. Chest.

[30] Kim H.C., Choi S.H., Huh J.W., Sung H., Hong S.B., Lim C.M., Koh Y. (2016). Different pattern of viral infections and clinical outcomes in patient with acute exacerbation of chronic obstructive pulmonary disease and chronic obstructive pulmonary disease with pneumonia.. J. Med. Virol..

[31] Lieberman D., Lieberman D., Gelfer Y., Varshavsky R., Dvoskin B., Leinonen M., Friedman M.G. (2002). Pneumonic *vs* nonpneumonic acute exacerbations of COPD.. Chest.

[32] Yu S., Fang Q., Li Y. (2018). Independent factors associated with pneumonia among hospitalized patients with acute exacerbations of chronic obstructive pulmonary disease.. Medicine.

[33] Kwok W.C., Tam T.C.C., Ho J.C.M., Lam D.C.L., Ip M.S.M., Yap D.Y.H. (2024). Hospitalized acute exacerbation in chronic obstructive pulmonary disease – impact on long-term renal outcomes.. Respir. Res..

[34] Basu R.K., Wheeler D.S. (2013). Kidney–lung cross-talk and acute kidney injury.. Pediatr. Nephrol..

[35] Doi K., Ishizu T., Fujita T., Noiri E. (2011). Lung injury following acute kidney injury: kidney–lung crosstalk.. Clin. Exp. Nephrol..

[36] Husain-Syed F., Slutsky A.S., Ronco C. (2016). Lung–Kidney Cross-Talk in the Critically Ill Patient.. Am. J. Respir. Crit. Care Med..

[37] Joannidis M., Forni L.G., Klein S.J., Honore P.M., Kashani K., Ostermann M., Prowle J., Bagshaw S.M., Cantaluppi V., Darmon M., Ding X., Fuhrmann V., Hoste E., Husain-Syed F., Lubnow M., Maggiorini M., Meersch M., Murray P.T., Ricci Z., Singbartl K., Staudinger T., Welte T., Ronco C., Kellum J.A. (2020). Lung–kidney interactions in critically ill patients: consensus report of the Acute Disease Quality Initiative (ADQI) 21 Workgroup.. Intensive Care Med..

[38] Kelly K.J. (2003). Distant effects of experimental renal ischemia/reperfusion injury.. J. Am. Soc. Nephrol..

[39] Andres-Hernando A., Dursun B., Altmann C., Ahuja N., He Z., Bhargava R., Edelstein C.E., Jani A., Hoke T.S., Klein C., Faubel S. (2012). Cytokine production increases and cytokine clearance decreases in mice with bilateral nephrectomy.. Nephrol. Dial. Transplant..

[40] Hepokoski M., Englert J.A., Baron R.M., Crotty-Alexander L.E., Fuster M.M., Beitler J.R., Malhotra A., Singh P. (2017). Ventilator-induced lung injury increases expression of endothelial inflammatory mediators in the kidney.. Am. J. Physiol. Renal Physiol..

[41] Yabuuchi N., Sagata M., Saigo C., Yoneda G., Yamamoto Y., Nomura Y., Nishi K., Fujino R., Jono H., Saito H. (2016). Indoxyl sulfate as a mediator involved in dysregulation of pulmonary Aquaporin-5 in acute lung injury caused by acute kidney injury.. Int. J. Mol. Sci..

